# A population-based study of homicide deaths in Ontario, Canada using linked death records

**DOI:** 10.1186/s12939-017-0632-9

**Published:** 2017-07-24

**Authors:** James Lachaud, Peter D. Donnelly, David Henry, Kathy Kornas, Andrew Calzavara, Catherine Bornbaum, Laura Rosella

**Affiliations:** 1 Dalla Lana School of Public Health, University of Toronto, 155 College Street, 6th Floor, Toronto, ON M5T 3M7 Canada; 2 Public Health Ontario, Santé publique Ontario, 480 University Avenue, Suite 300, Toronto, ON M5G 1V2 Canada; 3 Institute for Clinical Evaluative Sciences (ICES), G1 06, 2075 Bayview Avenue, Toronto, ON M4N 3M5 Canada; 4 Health & Rehabilitation Sciences, Faculty of Health Sciences, Western University, Elborn College, Room 2200, London, ON N6A 1H1 Canada

**Keywords:** Homicide, Socioeconomic status, Potential years of life lost, Canada

## Abstract

**Background:**

Homicide – a lethal expression of violence – has garnered little attention from public health researchers and health policy makers, despite the fact that homicides are a cause of preventable and premature death. Identifying populations at risk and the upstream determinants of homicide are important for addressing inequalities that hinder population health. This population-based study investigates the public health significance of homicides in Ontario, Canada, over the period of 1999–2012. We quantified the relative burden of homicides by comparing the socioeconomic gradient in homicides with the leading causes of death, cardiovascular disease (CVD) and neoplasm, and estimated the potential years of life lost (PYLL) due to homicide.

**Methods:**

We linked vital statistics from the Office of the Registrar General Deaths register (ORG-D) with Census and administrative data for all Ontario residents. We extracted all homicide, neoplasm, and cardiovascular deaths from 1999 to 2012, using International Classification of Diseases codes. For socioeconomic status (SES), we used two dimensions of the Ontario Marginalization Index (ON-Marg): material deprivation and residential instability. Trends were summarized across deprivation indices using age-specific rates, rate ratios, and PYLL.

**Results:**

Young males, 15–29 years old, were the main victims of homicide with a rate of 3.85 [IC 95%: 3.56; 4.13] per 100,000 population and experienced an upward trend over the study period. The socioeconomic neighbourhood gradient was substantial and higher than the gradient for both cardiovascular and neoplasms. Finally, the PYLL due to homicide were 63,512 and 24,066 years for males and females, respectively.

**Conclusions:**

Homicides are an important cause of death among young males, and populations living in disadvantaged neighbourhoods. Our findings raise concerns about the burden of homicides in the Canadian population and the importance of addressing social determinants to address these premature deaths.

**Electronic supplementary material:**

The online version of this article (doi:10.1186/s12939-017-0632-9) contains supplementary material, which is available to authorized users.

## Background

While violence is a key contributor to disease and disability [1, 2], homicide – a lethal expression of violence – has garnered less attention from health researchers and health policy makers [3, 4]. Compared to other causes of death, homicide rates remain relatively low in high income countries and have been largely reduced over the last decade, from 8.8 to 3.8 per 100,000 population [2]. In 2012, Canada, with a rate of 1.56 per 100,000 population [5], was ranked fifth for the highest homicide rate among 17 developed countries in the world. Homicide was classified as the 22nd cause of death in 2012 [6], and vital statistics showed that it represented 11.1% of all violent deaths in Canada over the 1992–2012 period.

Homicide mortality rates have remained relatively high among young people aged between 15 and 29 years old and young male adults between 30 and 44 years old [1, 2, 7]. In Canada, while the leading causes of death, neoplasm and heart disease, dominated the older age groups [8], homicide was the fourth leading cause of death for young people aged 1–24 years old, and the fifth leading cause for the 25–44 age group in 2012 [8]. As a consequence, the potential years of life lost (PYLL) are relatively high, as well as the associated economic costs, which include loss of income for the working years of life lost, costs for medical care, police services, and legal processes, and long periods of post-traumatic stress disorder for family members [9–11]. Another consequence of homicide is the fear generated at the community level, which may be a barrier to participation in health-promotive and social activities [12], and is associated with poorer mental health and reduced physical functioning [12, 13], as well as with population mobility and residential instability [14]. In other words, homicide has a negative effect on both individual sense of community and community cohesion [15].

Additionally, previous studies have shown that community, behavioral, and cross-cutting risk factors, such as frequent exposure to violent places where people excessively use or misuse alcohol and drugs [16, 17], interaction with those who have access to firearms [18–20], and regular use of violence to solve conflict [21, 22], played a key role in the victimization of homicide. A previous Canadian study [23] showed that neighbourhood income was a risk factor for homicide in that children less than 5 years old (and youth less than 15 years old) living in the lowest income neighbourhoods were 3.4 times (and 2.9 times) more likely to be a victim of homicide than their peers living in the wealthiest neighbourhoods. Hence, homicide may be an important contributor to health inequalities associated with the gap in life expectancy between the sexes and among socioeconomic groups [3, 6, 24, 25]. Accordingly, research examining homicide trends relative to socioeconomic (SES) may have key implications for policy development towards reducing these potential disparities in health.

This population-based study explores the patterns of homicides in Ontario, the most populous Canadian province, which has historically experienced the greatest number of homicides in Canada [26], with 2201 homicides recorded over the 1999–2012 period (see Table 1). We also compared the socioeconomic gradient in homicides with two leading causes of death in Ontario, cardiovascular and neoplasm deaths, and estimated the PYLL due to homicides.

**Table 1:** Characteristics of homicide, neoplasm, and cardiovascular disease (CVD) deaths that occurred in Ontario between 1999 and 2012

Characteristics	Homicide				Neoplasm				CVD			
	N	%	CI: 95%		N	%	CI: 95%		N	%	CI: 95%	
Age (means)	2201	(35.5)	(34.7	36.3)	355,882	(71.5)	(71.5	71.6)	373,952	(79.1)	(79.1	79.2)
% of females	662	30.08	28.2	33.6	170,248	47.8	47.7	48.1	188,672	50.5	50.3	50.7
Age group												
< 5	91	4.1	0.0	8.2	273	0.1	0.0	0.4	203	0.1	0.0	0.4
5–14	61	2.8	0.2	6.9	530	0.1	0.0	0.5	100	0.0	0.0	0.3
15–29	853	38.8	35.6	42.0	1653	0.5	0.2	0.8	729	0.2	0.0	0.5
30–44	563	25.6	23.3	29.2	9470	2.7	2.4	3.0	4907	1.3	1.0	1.6
45–64	458	20.8	18.3	24.5	85,223	23.9	23.7	24.2	42,787	11.4	11.2	11.7
≥ 65	175	8.0	5.8	12.0	258,733	72.7	72.6	72.9	325,226	87.0	86.9	87.1
Total	2201	100.0			355,882	100.0			373,952	100.0		

## Methods

### Data sources

We used data from the Institute for Clinical Evaluative Sciences (ICES), which linked vital statistics from the Office of the Registrar General Deaths register (ORG-D) with Census data for all Ontario residents. The ORG-D file contains all deaths registered in Ontario from January 1st 1990, including information on cause of death. Data on socioeconomic status were derived from Census years 2001 and 2006. These datasets were linked using unique encoded identifiers and analyzed at ICES.

### Outcomes variables

The primary outcome of this analysis was homicide deaths during the period 1999–2012 (inclusive). Homicide deaths were classified using the 3-digit International Classification of Diseases (ICD) code (ICD9: E960–969/ICD10: X85–X99, Y00–Y09, Y87.1) (*N* = 2201). We also extracted neoplasm deaths (ICD9: 140–208/ICD10: C00–C97) (*N* = 355,882), and cardiovascular deaths (ICD9: 390–398, 402, 404, 410–429/ICD10: I00–I09, I11, I13, I20–I51) (*N* = 373,952) to examine if variations exist in the socioeconomic gradient in homicide and these leading causes of death.

### Confounders

The potential confounders considered in this study were sex, age at time of death, and socioeconomic status (SES). Age at time of death was categorized as follows: <5, 5–14, 15–29, 30–44, 45–64, and ≥65 years old to facilitate comparison with previous studies [1, 2]. For SES, we used the Ontario Marginalization Index (ON-Marg) to measure two dimensions that contribute to the process of marginalization: material deprivation and residential instability [27]. ON-Marg is the Ontario adaptation of the Canadian Marginalization Index, which is used to measure inequalities in health, and is based on the dissemination area (DA), the smallest census area-level for which all census data are disseminated. ON-Marg has been previously validated for health research use in Ontario [28]. These ON-Marg indexes were only available for Census year 2001 and 2006. Therefore, this analysis was limited to decedents with dates-of-death between 1999 and 2012 (inclusive), where 2001 census-derived values were used for 1999–2003 and 2006 values for 2004–2012. Each ON-Marg dimension score was divided into quintiles, whereby quintile 1 corresponded to the least deprived (or residentially unstable) dissemination areas in Ontario and quintile 5 corresponded to the most deprived (or unstable) [27].

### Statistical analysis

We calculated age-specific death rates per 100,000 residents for both sexes by dividing the number of homicide deaths over the population of each age group by year and the combined 1999–2012 period. We computed the rate ratios by dividing rates of the first quintile of each ON-Marg dimension by the rates of the other quintiles. We estimated the PYLL due to homicide, a measure of premature mortality representing the total number of years not lived, by subtracting the age at death from the age limit commonly used to define premature deaths, 75 years old [29, 30], and summing the total PYLL according to sex and age group. All analyses were performed in SAS version 9.4.

### Ethics approval

The study obtained ethics approval from the Research Ethics Board at the University of Toronto and Sunnybrook Health Sciences Centre.

## Results

### Characteristics of homicide, neoplasm, and cardiovascular disease (CVD) deaths

Table 1 depicts the characteristics of the study population by cause of death. Homicide deaths had an average age of 35.5 years [95% CI: 34.7–36.3], while neoplasm and CVD deaths had an average age of 71.5 years [95% CI: 71.5–71.6], and 79.1 years [95% CI: 79.1–79.2], respectively. There were also marked sex differences among all three causes of death. Only 30% of homicides were among females, while about half of neoplasm and CVD deaths were among females.

### Sex-age pattern and trend of homicide deaths

**Fig. 1:**
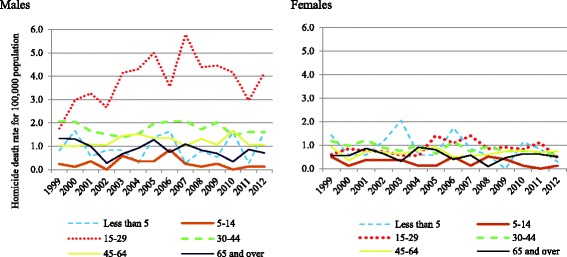
Trend in homicide mortality rates by age group per 100,000 population, in Ontario over the 1999–2012 period

Table 2 presents the age-specific homicide death rates in Ontario by sex over the 1999–2012 period. Homicide death rates were higher among males than females. In particular, young males between 15 and 29 years old were the primary victims of homicide with a rate of 3.85 per 100,000 population, followed by those between 30 and 44 years of age (1.77 per 100,000 population). Across all age groups, males had higher homicide rates than females, except for children aged 5 to 14 years where the homicide rate was lowest and similar for both sexes. Homicide mortality among young males (15–29 years) showed an upward trend over the 1999–2012 period (Fig. 1).

**Table 2:** Estimated age-specific homicide death rates per 100,000 population in Ontario, by age group, over the 1999–2012 period

Age group	Female				Male				Both sexes			
	N (%)	Rate	CI: 95%		N (%)	Rate	CI: 95%		N (%)	Rate	CI: 95%	
Less than 5	43 (6.5%)	0.93	0.72	1.15	48 (3.1%)	1.07	0.84	1.29	91 (4.1%)	1.00	0.84	1.16
5–14	30 (4.5%)	0.33	0.24	0.41	31 (2.0%)	0.29	0.21	0.37	61 (2.8%)	0.31	0.25	0.37
15–29	154 (23.3%)	0.94	0.83	1.06	699 (45.4%)	3.41	3.19	3.63	853 (38.8%)	2.19	2.06	2.32
30–44	188 (28.4%)	0.97	0.87	1.08	375 (24.4%)	1.90	1.75	2.05	563 (25.6%)	1.44	1.34	1.53
45–64	167 (25.2%)	0.76	0.67	0.86	291 (18.9%)	1.36	1.23	1.49	458 (20.8%)	1.06	0.98	1.14
65 and over	80 (12.1%)	0.56	0.46	0.66	95 (6.2%)	0.99	0.83	1.14	175 (8.0%)	0.75	0.66	0.84
Total	662 (100.0%)	0.77	0.01	0.01	1539 (100.0%)	1.70	0.02	0.02	2201 (100.0%)	1.23	1.19	1.28

### Homicide, cardiovascular, neoplasm deaths and socioeconomic status

**Fig. 2:**
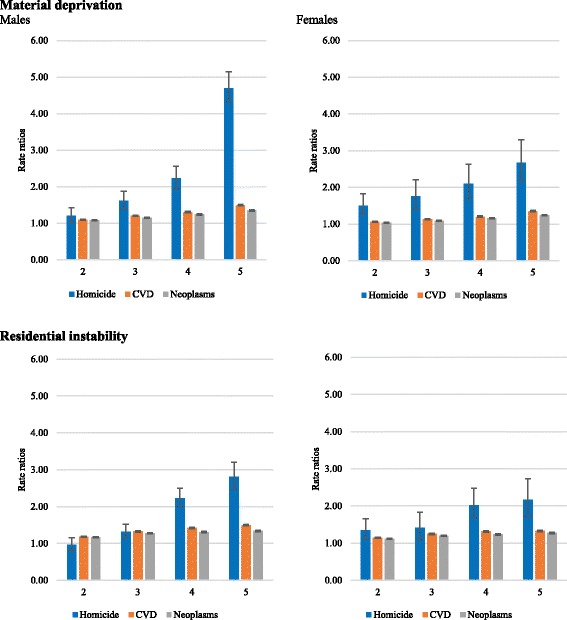
Homicide, cardiovascular disease (CVD), and cancer mortality rate ratios in Ontario by material deprivation and residential instability indices, 1999–2012

Figure 2 presents rate ratios of homicide, CVD, and neoplasm deaths relative to material deprivation and residential instability indices (see the full table of estimation in Additional file 1: Table S1). The results show that the mortality rate ratios for all three health outcomes were higher in the most materially deprived and residentially unstable neighbourhoods of Ontario. However, the neighbourhood material deprivation and residential instability gradients were higher for homicides, particularly among males, compared to the neighbourhood gradients observed for both CVD and neoplasm mortality.

### Homicide and PYLL

Table 3 shows the estimated PYLL due to homicides over the 1999–2012 period. In total, 87,578 years of life were estimated to have been lost in Ontario, with 63,512 years lost for males and 24,066 years lost for females. In addition, more than half of the PYLL were among youth aged 15–29 years old (44,893 years), and notably among young males (36,821 years).

**Table 3:** Estimated Potential Years of Life Lost (PYLL) by age group and socioeconomic status in Ontario over the period 1999–2012

	Male		Female		Both sexes	
	N	PYLL	N	PYLL	N	PYLL
Age group						
Less than 5	48	3554	43	3167	91	6721
5–14	31	2001	30	1963	61	3964
15–29	699	36,821	154	8072	853	44,893
30–44	375	14,352	188	7112	563	21,464
45–64	291	6493	167	3590	458	10,083
65+	95	291	80	162	175	453
Deprivation						
1	188	7512	99	3052	287	10,564
2	211	8332	127	4502	338	12,834
3	249	10,077	129	4629	378	14,706
4	292	12,020	132	4834	424	16,854
5	526	22,590	142	5786	668	28,376
Missing	73	2981	33	1263	106	4244
Instability						
1	235	10,015	105	3528	340	13,543
2	178	7400	108	4046	286	11,446
3	203	8219	97	3391	300	11,610
4	399	17,096	163	6196	562	23,292
5	451	17,801	156	5642	607	23,443
Missing	73	2981	33	1263	106	4244
Total PYLL	1539	63,512	662	24,066	2201	87,578

## Discussion

This study analyzed homicide deaths registered from 1999 to 2012 in Ontario, Canada from a population health perspective. The results showed that young males 15 to 29 years of age bore the burden of homicides, and that homicide death rates were relatively high among older males 30 to 44 years of age. The results also indicated that area-level marginalization factors relative to material deprivation and residential instability played a key role in homicide deaths in that homicide rate ratios were found to be higher in the most deprived and residentially unstable neighbourhoods of Ontario. Moreover, the socioeconomic neighbourhood gradient was higher for homicide deaths compared to both cardiovascular and neoplasm deaths. Finally, we found that the potential years of life lost were relatively high for males between 15 and 29 years of age in the most materially deprived and residentially unstable areas, suggesting that homicides are an important cause of premature death among young males living in the most disadvantaged neighbourhoods of Ontario.

The age-sex pattern of homicide deaths in high-income countries, such as Canada, is well documented in the violence literature [2, 7, 31]. Young males (15–29 years old) and young male adults (30–44 years old) have typically been the primary victims in all geographic regions during the last decade [1, 2, 31]. In addition, there is a consistent body of evidence that describes the association between socioeconomic deprivation and higher rates of homicides among groups with the least education [25, 31, 32], lower SES [31], and more deprived neighbourhoods [33, 34].

To our knowledge, no previous studies have compared the socioeconomic gradient in homicides with the leading causes of death. Our results revealed that socioeconomic-driven inequalities seem to be more pronounced in homicide than in both CVD and neoplasm deaths in Ontario over the period spanning 1999 to 2012. This may be explained by the preponderance of behavioral and cross-cutting risk factors in homicide compared to other causes of death [16, 18]. Moreover, our results suggest that homicide is a substantial contributor to sex and socioeconomic inequalities in health, particularly relative to potential years of life lost and, by extension, to life expectancy. A previous study from the United States [35] has shown a similar pattern in that homicide was a major cause of disparity in deaths among certain minority groups, including African Americans and non-Hispanic white groups. Frequent exposure to violence, alcohol misuse, drugs, and firearms have been largely discussed as risk factors for homicide in African American populations living in inner cities [20, 35, 36].

Although the victims of homicide are identified, there is a lack of research carried out on the consequences of homicide, particularly in relation to the health system. Previous studies showed that violence in general may have both behavioral and health consequences across the lifespan of the family and community members of the victims, including depression and anxiety, post-traumatic stress disorder, eating and sleep disorders, alcohol and drug abuse, and suicide [2, 9–11]. Further studies on the consequences of homicide on family and community members may be required to elucidate the full extent of the public health significance of homicide.

Our research findings are subject to a few limitations. The administrative data used provided limited variables for more complex analysis. Although the two dimensions of ON-Marg used provided neighbourhood socioeconomic measures at the smallest dissemination areas in the province, information on household and individual characteristics is not included, such as household income and behavioural risk factors. Finally, this research is limited to area-level assumptions.

## Conclusions

This paper argues that homicides are a key contributor to health inequalities as they disproportionally affect young males and those living in poorer neighbourhoods in Ontario, Canada. Our results suggest that homicides can provide important insights into population health inequities. Since homicides are an important cause of premature death among young males living in disadvantaged neighbourhoods, efforts to promote health by preventing homicides among young males in poor neighbourhoods present an important challenge for our health system.

## Additional file

Additional file 1: Table S1. Rates of homicide, neoplasm, and cardiovascular deaths per 100,000 population and rate ratios by sex and by material deprivation and residential instability indices. (DOCX 22 kb)

## Abbreviations

CI: Confidence interval
CVD: Cardiovascular disease
DA: Dissemination area
ICD: International Classification of Diseases
ICES: Institute for Clinical Evaluative Sciences
ON-Marg: Ontario Marginalization Index
ORG-D: Office of the Registrar General – Deaths
PYLL: Potential years of life lost
SES: Socioeconomic status
