# Supplementary material for: A population-based study of homicide deaths in Ontario, Canada using linked death records
Source: Int J Equity Health. 2017 Jul 24;16:133. doi: 10.1186/s12939-017-0632-9 (PMC5525348; doi:10.1186/s12939-017-0632-9)
Supplement: Additional file 1: Table S1. — Rates of homicide, neoplasm, and cardiovascular deaths per 100,000 population and rate ratios by sex and by material deprivation and residential instability indices. (DOCX 22 kb) [file 12939_2017_632_MOESM1_ESM.docx]

**Additional file 1**

Table S1: Rates of homicide, neoplasm, and cardiovascular deaths of undetermined intent per 100,000 population and rate ratios by sex and by material deprivation and residential instability indexes

| ON-Marg | Females | | | | | | | | | | | | | Males | | | | | | | | | | | | | | |
| --- | --- | --- | --- | --- | --- | --- | --- | --- | --- | --- | --- | --- | --- | --- | --- | --- | --- | --- | --- | --- | --- | --- | --- | --- | --- | --- | --- | --- |
|  | Homicide | | | | Cardiovascular | | | | | Neoplasms | | | | Homicide | | | | | Cardiovascular | | | | | Neoplasms | | | | |
|  | Rate | RR | IC: 95% | | Rate | RR | IC: 95% | | Rate | | RR | IC: 95% | | Rate | RR | IC: 95% | | Rate | | RR | IC: 95% | | Rate | | RR | IC: 95% | |  |
| Deprivation |  |  |  |  |  |  |  |  |  | |  |  |  |  |  |  |  |  | |  |  |  |  | |  |  |  |  |
| 1 | 0·4 | 1·00 | 0·76 | 1·32 | 109·2 | 1·00 | 0·99 | 1·01 | 125·3 | | 1·00 | 0·99 | 1·01 | 0·9 | 1·00 | 0·82 | 1·22 | 155·8 | | 1·00 | 0·99 | 1·01 | 163·0 | | 1·00 | 0·99 | 1·01 |  |
| 2 | 0·6 | 1·50 | 1·16 | 1·95 | 115·5 | 1·06 | 1·04 | 1·07 | 130·2 | | 1·04 | 1·02 | 1·05 | 1·1 | 1·21 | 0·99 | 1·47 | 170·9 | | 1·10 | 1·08 | 1·11 | 177·1 | | 1·09 | 1·07 | 1·10 |  |
| 3 | 0·7 | 1·76 | 1·35 | 2·28 | 123·4 | 1·13 | 1·11 | 1·15 | 136·7 | | 1·09 | 1·07 | 1·11 | 1·4 | 1·61 | 1·33 | 1·95 | 187·1 | | 1·20 | 1·18 | 1·22 | 188·0 | | 1·15 | 1·14 | 1·17 |  |
| 4 | 0·9 | 2·10 | 1·62 | 2·73 | 131·5 | 1·20 | 1·19 | 1·22 | 144·4 | | 1·15 | 1·13 | 1·17 | 2·0 | 2·23 | 1·86 | 2·68 | 203·2 | | 1·30 | 1·29 | 1·32 | 201·8 | | 1·24 | 1·22 | 1·26 |  |
| 5 | 1·1 | 2·67 | 2·07 | 3·45 | 148·1 | 1·36 | 1·34 | 1·38 | 155·1 | | 1·24 | 1·22 | 1·26 | 4·2 | 4·70 | 3·98 | 5·55 | 233·6 | | 1·50 | 1·48 | 1·52 | 220·5 | | 1·35 | 1·33 | 1·37 |  |
| Residential Instability |  |  |  |  |  |  |  |  |  | |  |  |  |  |  |  |  |  | |  |  |  |  | |  |  |  |  |
| 1 | 0·5 | 1·00 | 0·76 | 1·31 | 101·9 | 1·00 | 0·98 | 1·02 | 117·1 | | 1·00 | 0·98 | 1·02 | 1·1 | 1·00 | 0·83 | 1·20 | 144·7 | | 1·00 | 0·98 | 1·02 | 154·0 | | 1·00 | 0·98 | 1·02 |  |
| 2 | 0·6 | 1·35 | 1·03 | 1·77 | 116·5 | 1·14 | 1·12 | 1·16 | 131·2 | | 1·12 | 1·10 | 1·14 | 1·0 | 0·96 | 0·79 | 1·17 | 171·4 | | 1·18 | 1·17 | 1·20 | 179·8 | | 1·17 | 1·15 | 1·19 |  |
| 3 | 0·6 | 1·41 | 1·07 | 1·86 | 127·0 | 1·25 | 1·23 | 1·27 | 140·5 | | 1·20 | 1·18 | 1·22 | 1·4 | 1·32 | 1·09 | 1·59 | 191·6 | | 1·32 | 1·30 | 1·35 | 196·1 | | 1·27 | 1·25 | 1·29 |  |
| 4 | 0·9 | 2·02 | 1·58 | 2·58 | 134·5 | 1·32 | 1·30 | 1·34 | 144·7 | | 1·24 | 1·22 | 1·26 | 2·4 | 2·23 | 1·90 | 2·62 | 205·9 | | 1·42 | 1·40 | 1·44 | 201·3 | | 1·31 | 1·29 | 1·33 |  |
| 5 | 1·0 | 2·16 | 1·69 | 2·77 | 135·2 | 1·33 | 1·31 | 1·35 | 149·0 | | 1·27 | 1·25 | 1·29 | 3·0 | 2·81 | 2·40 | 3·29 | 216·8 | | 1·50 | 1·48 | 1·52 | 205·8 | | 1·34 | 1·32 | 1·36 |  |
